# Combined in ovo and post-hatch choline supplementation enhances growth and muscle development in goslings

**DOI:** 10.1016/j.psj.2026.107049

**Published:** 2026-05-01

**Authors:** Wenfeng Liu, Xucheng Zheng, Xuan Li, Haiming Yang, Zhiyue Wang

**Affiliations:** College of Animal Science and Technology, Yangzhou University, Yangzhou, Jiangsu Province 225009, PR China

**Keywords:** Choline supplementation, In ovo injection, Goslings, Growth performance, Muscle fiber morphology, Myogenic gene expression

## Abstract

This study evaluated how embryonic choline supplementation (ECS) via in ovo injection and gosling choline supplementation (GCS) via diet jointly influence embryonic development, endocrine status, growth performance, and muscle development. A total of 1,250 hatching eggs were allocated to five ECS treatments: non-injected control (E_NC_), sham-injected control (E_PC_), and 0.5 mL of choline chloride at 0.5 (E_0.5_), 1.0 (E_1_), and 1.5 (E_1.5_) mg/mL. Following hatch, 720 female goslings (144 per ECS group) were randomly assigned to three GCS levels: 0 (G_0_), 200 (G_200_), and 400 (G_400_) mg/kg.

ECS significantly increased embryo weight and relative embryo weight (embryo weight as a percentage of initial egg weight) at embryonic day 18 (DE18; *P* < 0.001) and DE27 (*P* = 0.005), while no significant effects were observed at DE20 (*P* > 0.05). Circulating growth hormone (GH), adrenocorticotropic hormone (ACT), and corticosterone (CORT) responded to choline in dose-dependent patterns (*P* < 0.001). Post-hatch body weight was significantly affected by the ECS × GCS interaction throughout the post-hatch period (*P* ≤ 0.016 at 7, 14, 21, and 28 days of age). GCS at 400 mg/kg significantly improved 28-day body weight (*P* < 0.001) and feed conversion ratio (*P* < 0.001). Pectoralis and leg muscle myofiber diameter and cross-sectional area were enhanced by higher choline supply (ECS: *P* ≤ 0.041; GCS: *P* < 0.001), accompanied by reduced pectoral fiber density (*P* < 0.001). Notably, myofiber hypertrophy occurred without changes in myogenin (MyoG), myogenic regulatory factor 4 (MRF4), or *myogenic factor 5 (Myf5)* expression (*P* > 0.05). Instead, choline exerted treatment-dependent modulation of *IGF-1* and *MSTN* primarily in the pectoralis (*P* < 0.001), indicating that choline promotes muscle growth via the IGF-1/MSTN axis. For growth rate and feed efficiency, E_1_G_400_ is preferable; for muscle yield, E_1.5_G_400_ offers additional fiber gains; for immune organ development, E_0_G_0_ is preferable given the increased bursa weights at lower supplementation. These findings provide a scientific basis for targeted choline nutritional strategies in goose production systems.

## Introduction

Geese are an important poultry species widely used for meat and egg production. However, relatively slow growth and suboptimal developmental performance, particularly during the early post-hatch period, constrain the expansion and efficiency of the goose industry. Compared with broiler chickens, which reach market weight (2.0-2.5 kg) within 35-42 days, geese exhibit relatively slow growth, with market weight (4.0-5.0 kg) typically achieved at 70-90 days of age. Therefore, improving embryonic development and subsequent growth of offspring remains a key challenge.

In animal cells, choline mainly exists as phosphatidylcholine, sphingomyelin, phosphocholine, acetylcholine, and glycerophosphocholine (Kenny et al., 2025), with most choline present in bound forms and only a small fraction as free choline (Burns et al., 2025). As a water-soluble, semi-essential nutrient, choline can be oxidized to betaine, contributing to osmotic regulation and one-carbon metabolism, and serves as the primary methyl donor for DNA, RNA, and protein methylation (Estrada-Cortés and Hansen, 2020). Because muscle growth requires intensive cell proliferation and protein synthesis, adequate methyl-donor supply is critical for myofiber development. In 1-day-old Ross 308 broiler chickens, supplementation with 1000 mg/kg choline improved feed conversion, growth performance, and pectoralis muscle protein content (Jahanian and Ashnagar, 2018).

In ovo feeding (IOF) is a technique in which nutrients are injected into the egg during incubation to enhance productive performance, overcoming limitations of conventional dietary strategies (Zielińska-Dawidziak et al., 2025). In ovo injection allows direct delivery of nutrients to the developing embryo, bypassing maternal metabolic conversion and variable deposition into eggs (Li et al., 2020). Compared with non-injected controls, chicks receiving in ovo nutrient injection showed greater late-stage body weight gain and improved feed conversion ratio, whereas simply increasing maternal dietary supply did not achieve comparable improvements (Bhattacharyya et al., 2017). In ovo injection of betaine (2.5 mg/egg) upregulated steroidogenic gene expression in chicken embryos, indirectly affecting muscle development (Abobaker et al., 2022). In geese, in ovo injection of 100 μL betaine increased pectoralis muscle weight by approximately 14% compared with the saline group, and both myofiber diameter and number were increased (Ma et al., 2024). Maternal choline supplementation increased pectoralis muscle fiber number and upregulated myogenic gene expression in broiler embryos (Gao et al., 2026), supporting the potential of direct embryonic choline delivery via IOF.

Muscle development is governed by specific transcriptional regulators. During embryogenesis, transcription factors such as *myogenic differentiation 1* (*MyoD*), *myogenic factor 5* (*Myf5*), *myogenin* (*MyoG*), and *myogenic regulatory factor 4* (*MRF4*) are key regulators of myoblast proliferation and differentiation. *MyoD* and *Myf5* initiate myogenic differentiation in early development, whereas *MyoG* promotes myofiber maturation and terminal differentiation (Conerly et al., 2016). In addition, *IGF-1* is a central anabolic factor in muscle development, stimulating myoblast proliferation and protein synthesis to promote muscle growth (Xun et al., 2025). Conversely, *myostatin* (*MSTN*) constrains excessive muscle growth and helps maintain normal muscle size and function (Lee et al., 2024). Although choline supplementation has been studied in laying hens and broilers (Beheshti Moghadam et al., 2021; Gao et al., 2026), its effects via in ovo administration, particularly in regulating offspring muscle growth in geese, remain insufficiently characterized.

We hypothesized that in ovo choline injection would enhance embryonic development and upregulate anabolic gene expression through methyl-donor-mediated epigenetic mechanisms; these embryonic priming effects would interact with post-hatch dietary choline to produce sustained improvements in growth performance; and the IGF-1/MSTN axis, rather than classical myogenic factors (*MyoG, MRF4, Myf5*), would be the primary pathway mediating choline-induced muscle hypertrophy. Accordingly, this study administered different concentrations of choline to goose embryos via IOF and assessed physiological indices during late incubation and the first 28 days post-hatch, with the goal of clarifying the role of combined embryonic and dietary choline in goose development and providing a scientific basis for optimizing choline supplementation strategies.

## Materials and methods

### Ethics statement

All animal care and experimental procedures in the study were performed according to the Regulations for the Administration of Affairs Concerning Experimental Animals of the People’s Republic of China and approved by the Animal Care and Use Committee of Yangzhou University, Yangzhou, China (202403131).

### Animals and sample collection

The experiment was conducted at the National Waterfowl Germplasm Resource Pool. Calcium dihydrogen phosphate, salt, DL-methionine, and premix feed were purchased from Yangzhou Yangda Stall Food Factory. The choline chloride (purity>99%, purchased from Saiguo Biotechnology Co., Ltd.) was converted to choline for supplement. We selected 1,250 Yangzhou goose hatching eggs, ensuring the female geese had similar egg-laying histories, parities, and nutrient intakes. Based on storage time (within 7 days), egg weight (144-154 g), and shape index (1.41-1.46), the eggs were divided into 5 treatment groups, 250 eggs each.

Embryonic choline supplementation (ECS) design was a one-factor test, while that for gosling choline supplementation (GCS) was a two-factor 4 × 3 trial. On day 8 of incubation, fertile eggs were identified by candling and subjected to different treatments: the control group (E_NC_) received no treatment, the blank group (E_PC_) was injected with 0.5 mL of preheated (37°C) deionized water, and the experimental groups (E_0.5_, E_1_, E_1.5_) were injected with 0.5 mL of choline chloride solution at concentrations of 0.5, 1, and 1.5 mg/mL, respectively. E_0_ designates the offspring derived from the E_NC_ group that received post-hatch dietary treatments. Post-hatching, 144 healthy female goslings from each treatment were selected, weighed, and randomly assigned to three dietary groups: G_0_ (no choline supplement), G_200_ (200 mg/kg choline), and G_400_ (400 mg/kg choline), as shown in Table 1.

**Table 1:** Outline of choline treatment paradigm.

Groups	Embyro choline supplement (mg/mL)	Gosling choline supplement (mg/kg)
E_NC_/E_0_	No treatment	/
E_PC_	0.5 mL deionized water/No choline	/
E_0.5_	0.5	/
E_1_	1	/
E_1.5_	1.5	/
E_0_G_0_	0	0
E_0_G_200_	0	200
E_0_G_400_	0	400
E_0.5_G_0_	0.5	0
E_0.5_G_200_	0.5	200
E_0.5_G_400_	0.5	400
E_1_G_0_	1	0
E_1_G_200_	1	200
E_1_G_400_	1	400
E_1.5_G_0_	1.5	0
E_1.5_G_200_	1.5	200
E_1.5_G_400_	1.5	400

The experimental feed samples were sent to the Beijing ZKGX Research Institute of Chemical Technology (Chemical Lab) for analysis. The actual content of choline in the diets was determined using ion chromatography (NY/T 1819-2009), while the actual content of methionine and cysteine in the diets was determined using the oxidative hydrolysis method (GB/T 18246-2019). The composition and nutritional concentration of the basic feed are shown in Table 2.

**Table 2:** Basal dietary composition and nutrient concentration (g/kg air-dried basic).

Items	Contents
Ingredient	
Corn	594.9
Soybean meal (430 g/kg CP)	290
Rice hull	30
Wheat bran	48
Lime stone	11
Calcium dihydrogen phosphate	10.5
Sodium chloride	3.00
DL-Methionine (990 g/kg)	2.1
L-lysine HCl	0.5
Vitamin and mineral premix	10.0
Nutritional components	
ME MJ/kg	11.5
CP	181
Crude fiber	43.0
Calcium	8.30
Total phosphorus	6.30
Nonphytate phosphorus	3.80
Lysine	9.90
Methionine	4.80
Cystine	3.22
Methionine+Cystine	8.02
Arginine	12.5
^5^Choline (mg/kg)	328

Note: The premix supplied per kilogram of diet: vitamin A, 12000 IU; vitamin D_3_, 4000 IU; vitamin E, 28 mg; vitamin K_3_, 1.5 mg; vitamin B_1_, 0.9 mg; vitamin B_2_, 8 mg; vitamin B_6_, 3.2 mg; vitamin B_12_, 0.01 mg; nicotinic acid, 45 mg; pantothenic acid, 11 mg; folic acid, 0.65 mg; biotin, 0.05 mg; Fe, 60 mg; Cu, 10 mg; Mn, 95 mg; Zn, 90 mg; I, 0.5 mg; Se, 0.3 mg. The values of methionine, cystine, choline are measured value, other values are calculated values. The mesured values of methionine for treatments were 4.80 g/kg, 4.85 g/kg, 4.80 g/kg, 4.75 g/kg, 4.85 g/kg, 4.90 g/kg, 4.80 g/kg, 4.85 g/kg, 4.70 g/kg, 4.75 g/kg, 4.75 g/kg, 4.70 g/kg, 4.90 g/kg, 4.90 g/kg and 4.70 g/kg. The mesured values of cystine for treatments were 3.22 g/kg, 3.30 g/kg, 3.25 g/kg, 3.10 g/kg, 3.30 g/kg, 3.25 g/kg, 3.30 g/kg, 3.25 g/kg, 3.10 g/kg, 3.15 g/kg, 3.10 g/kg, 3.20 g/kg, 3.35 g/kg, 3.20 g/kg and 3.15 g/kg. The mesured values of choline for treatments were 328, 510, and 755 mg/kg, respectively.

Hatching was performed using the Keyou CFZ microcomputer-controlled fully automatic incubator from Dezhou Keyou Hatching Equipment Co., Ltd. The incubation period was divided into three stages based on the embryonic development of geese: early (days 1-14), middle (days 15-28), and late (days 29-30). A variable-temperature regime was used for the entire batch. The temperature and humidity were controlled at 37.8°C and 70% in the early stage, 37.5°C and 65% in the middle stage, and 36.5°C and 72% in the late stage. From day 18 to day 24 of incubation, eggs were cooled once daily at noon. From day 25 to day 28, eggs were cooled three times daily. On day 29 of incubation, eggs were transferred to a hatcher for hatching. Other aspects of incubation management followed the conventional hatching procedure. Egg injection followed the method of Tako et al. (2004) with modifications. First, locate the yolk sac via candling and disinfect the blunt end with 75% alcohol. Drill a proper-sized hole and inject 0.5 ml of nutrient solution into the yolk sac vertically with a 2-cm-deep needle, using a new needle for each egg. Then disinfect again, seal the hole with sterile paraffin wax, and place the eggs in the incubator, distributing them evenly to minimize layer-to-layer effects.

The gosling choline supplementation experiment lasted for 28 days. For the 1 to 5-day-old goslings, the temperature was maintained at 28-30°C. For the 21 to 30-day-old goslings, it was adjusted to 22-18°C, and for the 6 to 20-day-old goslings, it was reduced by 2°C every 5 days. The temperature in the rearing environment for gosling stages was kept between 10 and 25°C. During the brooding period, the relative humidity was adjusted to 60%−75%, and for gosling stages, a humidity of 50%−65% was maintained. For 1-day-old goslings, the temperature in the coop was maintained at 30-32°C, and as they grew older, it was lowered by 0.5°C daily. After 15 days, the temperature was kept between 18°C and 20°C. The light time and night lamp power of the goslings at 1, 2 and 3 weeks old were 24-23 hours, 22-18 hours and 18-14 hours respectively, and the night lamp power was 15W/100 square meters. Based on the nutritional requirements for geese recommended (NRC, 1994), the feed was formulated, and the goslings were divided into different treatments for rearing. They were given water within 12 h of hatching and food within 24 h, with strict control over the rearing environment conditions. The management and immunization of the goslings were carried out according to normal procedures. Post-hatch, hatchability was calculated by replicate, excluding dead embryos detected via candling before egg injection. Daily feed intake was recorded, and body weight was measured at 0, 7, 14, 21, and 28 days post-hatch, based on replicates. The average daily feed intake (ADFI), average daily gain (ADG), and feed-to-gain ratio (F/G) were calculated. At 28 days post-hatch, body weight (BW) and body measurements were recorded for each gosling. The following body measurements were taken using a caliper or ruler (accurate to 0.1 cm): semi-submerged length, neck length, body length, keel length, tibia circumference, chest width, chest depth, hip width, and tibia length. Semi-submerged length was measured using a flexible measuring tape by two trained observers and defined as the surface contour distance from the upper edge of the bill, along the neck and back, to the midpoint of the hip bone; the mean of duplicate readings was recorded.

The following organ weights were measured after dissection: heart, liver, spleen, kidney, thymus, bursa of Fabricius, pectoralis muscle, and leg muscle. Organs were carefully excised, trimmed of excess tissue, and weighed using an analytical balance (accurate to 0.001 g). The organ indices were calculated by dividing the organ weight (in grams) by the body weight (in grams) and multiplying by 100.

At embryonic day 23 and 27, the yolk sac was weighed after dissection. Tissues were sampled from 6 goslings/embryos per group at embryonic days 18, 20, 23, 27, and 28 days post-hatch. At embryonic days 23 and 27, and at 28 days post-hatch, the left pectoralis and leg muscle samples were randomly collected from 6 goslings per group. All samples were rapidly frozen in liquid nitrogen and stored at −80°C for mRNA expression analysis. To analyze the expression of muscle development-related genes, real-time quantitative PCR (qPCR) was used to measure the mRNA levels of *MyoG, Myf5, MRF4, MSTN*, and *IGF-1*.

The right pectoralis and leg muscles were weighed, fixed in paraformaldehyde for HE staining, and used for myofiber morphological analysis. Samples from paraformaldehyde-fixed muscles were processed into paraffin sections, stained with HE, and observed under an optical microscope (IX73, Olympus, Japan) and a Toup View video imaging system at 10 × 10 magnification. For each image, a random field was selected.

Blood samples were taken from the jugular vein of goslings at 7, and 28 d. Blood samples were collected from the jugular vein of goslings at 7, and 28 days of age into serum separator tubes. The samples were centrifuged at 3000 × *g* for 15 min, and the supernatants were aliquoted into 0.5 mL EP tubes and stored at −20°C for analysis of adrenocorticotropic hormone (ACTH), corticosterone (CORT), activin (ACT) levels. Goslings were euthanized after blood collection.

### Measurement of serum reproductive hormones

Serum concentrations of ACT, CORT, IGF-1, T_3_, and T_4_ were measured using a one-step sandwich ELISA with a double antibody kit from Elabscience. The antigens were conjugated with Keyhole Limpet Hemocyanin (KLH), and the antibodies used was rabbit monoclonal antibodies for IGF-1, and mouse monoclonal antibodies for GH, CORT, ACT, T_3_, and T_4_.

### Expression of gonadotropin-releasing hormone mRNA in the muscle

Total RNA isolation, cDNA synthesis, and quantitative PCR were performed according to the methods described in a previously published paper (Osman et al., 2016). The specificity of the primers was confirmed by melting curve analysis. The reference gene was GAPDH, and the primer sequences for each gene are listed in Table 2. The 2^-ΔΔCt^ method was used to calculate the relative expression levels of the target genes (Table 3).

**Table 3:** Sequences of the primers for the target genes.

Target Genes	Primer sequences (5′−3′)	Size/bp	Accession No.
*MyoG*	F: CGCCGCCTGAAGAAGGTGAA	154	XM_048070946.1
	R: CCTGCTGGTTGAGGGTGCTGA		
*MRF4*	F: GGTTGTTCCTCGGGGTGTTTTC	127	XM_048078889.1
	R: CTCCTTCTCCCCGTCCAAGTAG		
*Myf5*	F:AGCTCTTGAGGGAGCAGGTA	175	XM_048078888.1
	R:GTACCCGTGGGGCATCTC		
*IGF-1*	F:TACCTTGGCCTGTGTTTGCT	170	XM_013181061.2
	R:CCCTTGTGGTGTAAGCGTCT		
*MSTN*	F:AGTTGATCCGGTGGCTCTTG	162	NM_001001461.2
	R:TTAGGAGCTTGTTCCAGGCG		
*GAPDH*	F:GCTGATGCTCCCATGTTCGTGAT	86	DQ821717.1
	R:GTGGTGCAAGAGGCATTGCTGAC		

### Statistical analysis

**Fig. 1:**
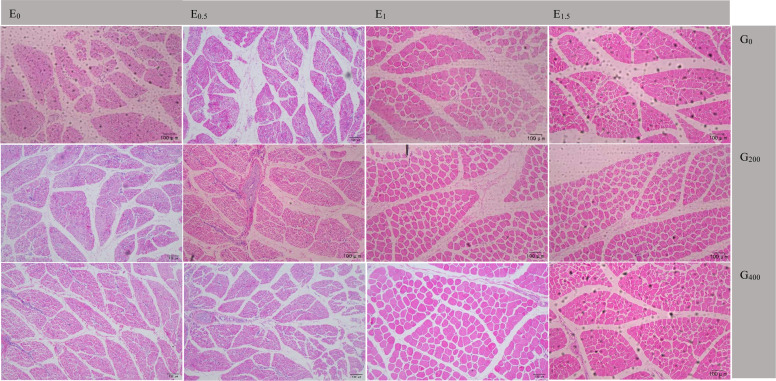
Representative H&E-stained sections of pectoralis muscle from 28-day-old goslings subjected to embryonic and dietary choline supplementation.

**Fig. 2:**
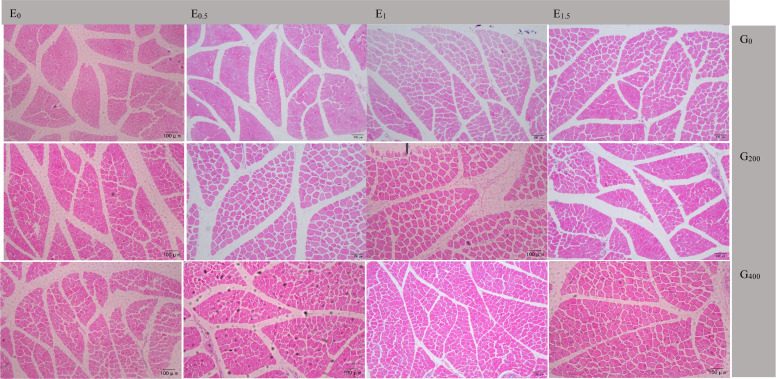
Representative H&E-stained sections of leg muscle from 28-day-old goslings subjected to embryonic and dietary choline supplementation.

Note: Representative images were captured at 10 × 10 magnification. Columns indicate embryonic choline supplementation levels (0, 0.5, 1, 1.5 mg/ml), and rows indicate dietary choline levels in offspring diets (0, 200, 400 mg/kg). Scale bar = 100 μm.

Microsoft Excel 2019 (v. 1808, MS, Redmond, WA, USA) was used for preliminary data processing, while Two-way ANOVA, polynomial contrast (linear, quadratic), and bivariate correlation analysis were conducted using SPSS 19.0 statistical software (Chicago, IL, USA). Treatment comparisons were performed using Tukey's multiple range tests. Given the large number of statistical comparisons conducted across this study, all P values were adjusted for multiple comparisons using the Benjamini-Hochberg false discovery rate (FDR) procedure, with the FDR controlled at 0.05. Statistical significance was declared when FDR-adjusted *P* < 0.05. Data were presented as mean and pooled standard error of the mean (SEM) (Figs. 1 and 2).

Note:Representative images were captured at 10 × 10 magnification. Columns indicate embryonic choline supplementation levels (0, 0.5, 1, 1.5 mg/ml), and rows indicate dietary choline levels in offspring diets (0,200,400 mg/kg). Scale bar = 100 μm.

## Results

### In ovo choline feeding enhances embryonic growth and hormonal profiles

According to Table 4, ECS significantly affected embryo weight and relative embryo weight at DE18 (*P* < 0.001) and DE27 (*P* = 0.005 and *P* = 0.020, respectively), but not at DE20 (*P* > 0.05). At DE23, relative embryo weight responded to ECS (*P* = 0.047). Quadratic effects on both parameters were significant at DE18 (*P* < 0.001), whereas linear effects were not.

**Table 4:** Effects of in ovo choline on embryo weight and relative embryo weight.

Age(d)	Items	Treatments					SEM	*P*-value		
		E_NC_	E_PC_	E_0.5_	E_1_	E_1.5_		choline	linear	quadratic
DE18	Embryo weight(g)	18.53^ab^	14.61^c^	15.57^bc^	20.24^a^	19.03^a^	0.513	<0.001	0.640	<0.001
	Relative weight of embryo(g/g)	0.1277^ab^	0.1017^c^	0.1071^bc^	0.1364^a^	0.1266^b^	0.003	<0.001	0.710	0.013
DE20	Embryo weight(g)	26.13	23.79	24.18	24.48	24.85	0.371	0.444	0.426	0.839
	Relative weight of embryo(g/g)	0.1791	0.1634	0.1664	0.1676	0.1695	0.002	0.412	0.432	0.937
DE23	Embryo weight(g)	43.49	42.42	46.43	45.30	45.69	0.890	0.431	0.640	0.335
	Relative weight of embryo(g/g)	0.3027^ab^	0.2775^b^	0.3300^a^	0.3143^ab^	0.3281^ab^	0.006	0.047	0.260	0.228
DE27	Embryo weight(g)	70.65^b^	70.03^b^	74.13^a^	75.41^a^	76.66^a^	0.981	0.005	0.335	0.222
	Relative weight of embryo(g/g)	0.4665^b^	0.4762^b^	0.5078^ab^	0.5325^a^	0.5403^a^	0.007	0.020	0.556	0.335

Note: DE18, DE20, DE23 and DE27 indicate days of embryonic development. Treatments: E_NC_ = non-injected control; E_PC_ = sham-injected control (0.5 mL prewarmed deionized water); E_0.5_, E_1_, and E_1.5_ = in ovo injection of 0.5 mL choline chloride solution at 0.5, 1.0, and 1.5 mg/mL, respectively. Relative weight of embryo (g/g) = embryo weight / egg weight. SEM = standard error of the mean. Means within the same row with different superscript letters (a–c) differ significantly (*P* < 0.05, FDR-adjusted). The *P*-value columns indicate the overall choline effect and the linear and quadratic trends based on FDR-adjusted *P* values.

Results presented in Table 5 show that ECS significantly affected GH, ACT, and CORT at DE18 (*P* ≤ 0.006), DE20 (*P* < 0.001), and DE23 (*P* ≤ 0.020). GH and ACT showed linear responses at DE18 (*P* ≤ 0.011), whereas CORT did not (*P* = 0.527). Only GH showed a linear response at DE20 (*P* = 0.008). At DE23, all three hormones displayed linear responses (*P* ≤ 0.005). Quadratic effects were significant at DE18 for all indicators (*P* < 0.001), at DE20 for GH, ACT, and CORT (*P* < 0.001), and at DE23 for ACT and CORT (*P* ≤ 0.045), but not for GH (*P* > 0.05).

**Table 5:** Effects of in ovo choline supplementation on circulating hormones in goose embryos.

Age (d)	Items	Treatments					SEM	*P*-value		
		E_NC_	E_PC_	E_0.5_	E_1_	E_1.5_		choline	linear	quadratic
DE18	GH (ng/ml)	10.85^b^	11.33^b^	13.44^a^	14.40^a^	11.51^b^	0.301	<0.001	0.011	<0.001
	ACT (ng/ml)	35.21^a^	32.74^ab^	27.00^b^	30.61 ^ab^	35.49^a^	0.850	0.006	<0.001	<0.001
	CORT (pg/ml)	393.0^a^	383.8^ab^	306.8^c^	349.5^b^	399.6a	7.274	<0.001	0.527	<0.001
DE20	GH (ng/ml)	10.31^c^	11.29^bc^	12.84^ab^	14.83^a^	11.06^bc^	0.359	<0.001	0.008	<0.001
	ACT (ng/ml)	34.43^a^	29.18^bc^	25.50^c^	29.30^bc^	32.49^ab^	0.729	<0.001	0.411	<0.001
	CORT (pg/ml)	382.5a	294.9b	282.3b	329.8ab	361.3a	9.295	<0.001	0.893	<0.001
DE23	GH (ng/ml)	10.85^b^	11.15^b^	13.89^a^	15.48^a^	15.09^a^	0.418	<0.001	<0.001	0.312
	ACT (ng/ml)	29.77^a^	28.89^ab^	24.97^cd^	24.42^d^	26.90^bc^	0.454	<0.001	<0.001	<0.001
	CORT (pg/ml)	343.4^a^	317.0^ab^	297.3^b^	291.8^b^	300.9^b^	5.375	0.020	0.005	0.045

Note: DE18, DE20 and DE23 indicate days of embryonic development. Treatments: E_NC_ = non-injected control; E_PC_ = sham-injected control (0.5 mL prewarmed deionized water); E_0.5_, E_1_, and E_1.5_ = in ovo injection of 0.5 mL choline chloride solution at 0.5, 1.0, and 1.5 mg/mL, respectively. SEM = standard error of the mean. Means within the same row with different superscript letters (a–d) differ significantly (*P* < 0.05, FDR-adjusted). The *P*-value columns indicate the overall choline effect and the linear and quadratic trends based on FDR-adjusted *P* values. Abbreviations: GH, growth hormone; ACT, adrenocorticotropic hormone; CORT, corticosterone.

### In ovo choline supplementation enhances embryonic development and growth in goslings

#### In ovo choline effects on gosling body weight and growth performance

Table 6 indicates that, at 0 d, BW was significantly affected by ECS (*P* = 0.022) and ECS × GCS interaction (*P* < 0.001), but not by GCS (*P* = 0.692). BW in E0 was lower than in other groups. From 7 to 21 d, ECS, GCS, and their interaction significantly affected BW (*P* < 0.001), with E1 and G400 generally showing the highest values; E_1_G_400_ achieved the highest BW at 21 d (1208 g). At 28 d, GCS (*P* < 0.001) and the ECS × GCS interaction (*P* < 0.001), but not ECS (*P* = 0.831), significantly affected BW. Significant ECS × GCS interactions were observed at all time points (*P* = 0.016).

**Table 6:** Effect of different choline supplementation concentrations in the embryo and its offspring diets on body weight of goslings.

Items	ECS (mg/kg)	GCS (mg/kg)	0d BW (g)	7d BW (g)	14d BW (g)	21d BW (g)	28d BW (g)
E_0_G_0_	0	0	101.5	206.4	519.1	1052	1460
E_0_G_200_	0	200	101.8	212.0	533.3	1017	1560
E_0_G_400_	0	400	101.1	206.5	629.0	1167	1690
E_0.5_G_0_	0.5	0	103.0	178.9	587.2	1072	1540
E_0.5_G_200_	0.5	200	102.6	190.5	596.0	1145	1577
E_0.5_G_400_	0.5	400	102.8	226.9	596.0	1122	1677
E_1_G_0_	1.0	0	102.8	196.4	577.7	1042	1401
E_1_G_200_	1.0	200	102.9	231.7	591.8	1177	1583
E_1_G_400_	1.0	400	102.5	220.1	652.1	1208	1840
E_1.5_G_0_	1.5	0	102.5	196.8	495.6	917.8	1425
E_1.5_G_200_	1.5	200	103.1	214.2	609.9	1104	1517
E_1.5_G_400_	1.5	400	102.7	207.1	596.1	1158	1763
SEM			1.176	1.405	3.990	5.935	9.434
	0		101.5^b^	198.8^b^	559.5^c^	1081^b^	1578
	0.5		102.8^a^	208.4^ab^	593.5^ab^	1111^ab^	1588
	1		102.7^a^	216.0^a^	608.1^a^	1150^a^	1594
	1.5		102.8^a^	206.1^b^	569.8^bc^	1069^b^	1588
		0	102.3	194.6^b^	545.3^c^	1026^c^	1458^c^
		200	102.7	212.1^a^	580.6^b^	1110^b^	1561^b^
		400	102.3	215.2^a^	618.0^a^	1164^a^	1741^a^
*P*-value	ECS		0.022	0.000	0.000	0.000	0.831
	GCS		0.692	0.000	0.000	0.000	0.000
	ECS.GCS interaction		0.000	0.000	0.016	0.000	0.000

Note: ECS indicates embryonic choline supplementation level (in ovo injection; mg/mL), and GCS indicates dietary choline supplementation level in offspring diets (mg/kg). Treatments are expressed as combinations (e.g., E_0_G_0_, E_0.5_G_200_, E_1.5_G_400_). BW = body weight; *d* = day(s) of age. SEM = standard error of the mean. For the main-effect means (ECS or GCS), values within the same column with different superscript letters (a-c) differ significantly (*P* < 0.05, FDR-adjusted). *P*-values indicate the effects of ECS, GCS, and their interaction (ECS × GCS) based on FDR-adjusted *P* values.

Table 7 indicates that ADFI during 1–7 d and 8–14 d was not significantly affected by ECS, GCS, or their interaction (*P* > 0.05). During 1–7 d, ADG was significantly affected by GCS (*P* = 0.003), with G_400_ showing the highest ADG, whereas ECS and the interaction had no significant effects (*P* > 0.05). For F/G, ECS (*P* < 0.001) and GCS (*P* < 0.001) showed significant main effects, with E_0_ having the highest F/G and G_400_ the lowest. During 8–14 d, GCS significantly affected ADG and F/G (*P* ≤ 0.049), while ECS and the interaction were not significant (*P* > 0.05). During 15–21 d, ECS significantly affected ADFI and F/G (*P* ≤ 0.026), and GCS significantly affected ADG and F/G (*P* ≤ 0.020), with G_400_ showing the lowest F/G. Significant interactions were observed for ADFI and F/G (*P* ≤ 0.041). During 22–28 d, ECS and GCS significantly affected ADFI, ADG, and F/G (*P* < 0.001), with interactions remaining significant for ADFI and F/G (*P* ≤ 0.049).

**Table 7:** Effect of different choline supplementation concentrations in the embryo and its offspring diets on growth performance of goslings.

Items	ECS (mg/kg)	GCS (mg/kg)	1-7 d			8-14 d			15-21 d			22-28 d		
			ADFI (g)	ADG (g)	F/G (g/g)	ADFI (g)	ADG (g)	F/G (g/g)	ADFI (g)	ADG (g)	F/G (g/g)	ADFI (g)	ADG (g)	F/G (g/g)
E_0_G_0_	0	0	36.88	15.24	2.42	115.38	54.23	2.13	174.7	66.95	2.61	173.7	69.77	2.49
E_0_G_200_	0	200	39.66	17.47	2.27	86.49	50.06	1.73	154.2	73.57	2.10	169.3	70.69	2.39
E_0_G_400_	0	400	36.00	16.00	2.25	96.10	57.20	1.68	161.0	77.18	2.09	131.0	72.86	1.80
E_0.5_G_0_	0.5	0	34.24	14.57	2.35	106.6	53.85	1.98	165.8	67.16	2.47	182.4	71.20	2.56
E_0.5_G_200_	0.5	200	31.32	14.64	2.14	87.14	50.08	1.74	137.1	69.77	1.97	122.3	73.60	1.66
E_0.5_G_400_	0.5	400	41.04	19.18	2.14	93.07	55.73	1.67	131.3	76.88	1.71	131.4	82.24	1.60
E_1_G_0_	1.0	0	35.45	13.48	2.63	92.37	47.86	1.93	125.1	59.00	2.12	97.32	61.60	1.58
E_1_G_200_	1.0	200	45.06	18.62	2.42	98.62	57.67	1.71	128.9	69.06	1.87	107.8	69.14	1.56
E_1_G_400_	1.0	400	36.57	17.84	2.05	99.06	61.53	1.61	124.4	82.14	1.51	119.8	78.79	1.52
E_1.5_G_0_	1.5	0	44.15	13.71	3.22	90.10	48.18	1.87	102.3	53.03	1.93	165.5	65.12	2.54
E_1.5_G_200_	1.5	200	39.10	14.98	2.61	93.65	56.08	1.67	124.3	69.84	1.78	192.8	85.16	2.26
E_1.5_G_400_	1.5	400	31.30	15.57	2.01	100.1	58.89	1.70	138.9	80.74	1.72	136.1	84.52	1.61
SEM			1.118	0.412	0.11	7.895	1.183	0.15	6.702	1.272	0.18	6.772	1.261	0.19
	0		37.51	16.30	2.31^b^	99.07	53.83	1.84	164.7^a^	72.57	2.27^a^	158.6^a^	71.11	2.23^a^
	0.5		35.54	16.14	2.21^c^	95.26	53.22	1.79	146.1^b^	71.26	2.05^ab^	146.8^b^	75.68	1.94^b^
	1		39.03	16.78	2.37^b^	97.46	55.69	1.75	128.2^c^	70.07	1.83^b^	108.3^c^	69.85	1.55^c^
	1.5		38.18	14.83	2.61^a^	94.45	54.38	1.74	122.8^c^	67.87	1.81^b^	166.7	78.27	2.13^b^
		0	37.68	14.18^b^	2.66^a^	101.0^a^	51.03^b^	1.98^a^	140.3	61.52^c^	2.28^a^	153.2	66.92^b^	2.29^a^
		200	38.79	16.51^a^	2.36^b^	91.45^b^	53.48^ab^	1.71^b^	136.2	70.56^b^	1.93^b^	146.3	74.65^a^	1.96^b^
		400	36.23	17.15^a^	2.11^c^	97.43^b^	58.34^a^	1.67^b^	139.4	79.23^a^	1.76^bc^	129.7	79.60^a^	1.63^c^
*P*-value	ECS		0.426	0.305	<0.001	0.677	0.896	0.863	<0.001	0.693	0.026	<0.001	0.120	<0.001
	GCS		0.501	0.003	<0.001	0.457	0.047	0.049	0.426	<0.001	0.020	0.221	0.003	<0.001
	ECS.GCS interaction		0.591	0.418	0.051	0.896	0.553	0.933	0.035	0.455	0.041	0.022	0.374	0.049

Note: ECS indicates embryonic choline supplementation level (in ovo injection; mg/mL), and GCS indicates dietary choline supplementation level in offspring diets (mg/kg). Treatments are expressed as combinations (e.g., E_0_G_0_, E_0.5_G_200_, E_1.5_G_400_). ADFI = average daily feed intake; ADG = average daily gain; F/*G* = feed-to-gain ratio; *d* = day(s) of age. SEM = standard error of the mean. For the main-effect means (ECS or GCS), values within the same column with different superscript letters (a-c) differ significantly (*P* < 0.05, FDR-adjusted). *P*-values indicate the effects of ECS, GCS, and their interaction (ECS × GCS) based on FDR-adjusted *P* values.

#### In ovo choline effects on gosling morphology and organ development

Table 8 indicates that, at 28 d, BW, semi-submerged length, and keel length were significantly affected by GCS (*P* < 0.05), with these traits higher in G_400_ than in G_0_ and G_200_. GCS showed no significant effects on neck length, body length, tibia circumference, chest width, chest depth, hip width, or tibia length (*P* > 0.05). ECS did not significantly influence any morphometric traits (*P* > 0.05). Significant ECS × GCS interactions were observed for BW, tibia circumference, and hip width (*P* < 0.05).

**Table 8:** Effect of different choline supplementation concentrations in the embryo and its offspring diets on body weight and body size traits of goslings at 28 days of age.

Items	ECS (mg/kg)	GCS (mg/kg)	BW (kg)	Semi-Submerged Length (cm)	Neck Length(cm)	Body Length(cm)	Keel Length(cm)	Tibia Circumference(cm)	Chest Width(cm)	Chest Depth(cm)	Hip Width(cm)	Tibia Length(cm)
E_0_G_0_	0	0	1.61	36.28	13.00	20.27	14.45	4.40	4.75	3.48	5.97	14.43
E_0_G_200_	0	200	1.62	36.62	13.79	20.10	16.43	4.27	4.25	4.15	5.50	14.07
E_0_G_400_	0	400	1.54	36.40	13.20	20.20	18.09	3.92	4.40	3.50	5.10	14.54
E_0.5_G_0_	0.5	0	1.56	36.36	13.52	20.02	14.46	4.14	4.40	3.60	5.50	14.32
E_0.5_G_200_	0.5	200	1.40	36.37	13.30	19.81	15.82	4.05	4.41	4.33	5.50	14.15
E_0.5_G_400_	0.5	400	1.76	37.90	14.00	20.52	18.17	4.40	4.90	3.90	5.90	14.62
E_1_G_0_	1.0	0	1.35	35.35	12.38	21.80	15.31	3.83	4.82	4.00	5.13	14.43
E_1_G_200_	1.0	200	1.63	37.80	13.45	20.20	16.93	4.25	4.58	3.87	5.75	14.40
E_1_G_400_	1.0	400	1.73	39.05	13.35	21.95	17.25	4.25	4.83	4.25	5.75	15.03
E_1.5_G_0_	1.5	0	1.32	36.24	13.12	18.99	15.15	3.94	4.20	3.76	4.97	13.60
E_1.5_G_200_	1.5	200	1.52	36.50	12.35	20.50	16.93	4.03	5.00	3.75	5.33	14.30
E_1.5_G_400_	1.5	400	1.85	38.08	12.66	20.04	17.47	4.18	4.20	4.38	5.40	14.92
SEM			0.02	0.269	0.156	0.208	0.134	0.029	0.098	0.194	0.158	0.905
	0		1.59	36.45	13.33	20.18	16.32	4.19	4.47	3.38	5.52	14.35
	0.5		1.57	36.88	13.60	20.11	16.15	4.20	4.57	3.61	5.63	14.36
	1		1.57	36.94	13.05	21.31	16.50	4.11	4.74	4.03	5.54	14.62
	1.5		1.56	37.40	12.71	19.84	16.51	4.05	4.47	3.96	5.23	14.27
		0	1.46^b^	36.05^b^	13.00	20.27	14.83^c^	4.07	4.54	3.71	5.39	14.20
		200	1.54^b^	36.82^ab^	13.22	20.15	16.66^b^	4.15	4.56	3.76	5.52	14.23
		400	1.72^a^	37.87^a^	13.30	20.67	17.61^a^	4.19	4.58	3.78	5.54	14.78
*P*-value	ECS		0.993	0.723	0.335	0.723	0.800	0.335	0.499	0.100	0.169	0.710
	GCS		<0.001	0.045	0.778	0.549	<0.001	0.416	0.570	0.963	0.620	0.120
	ECS.GCS interaction		0.011	0.659	0.657	0.581	0.106	<0.001	0.137	0.258	0.018	0.751

Note: ECS indicates embryonic choline supplementation level (in ovo injection; mg/mL), and GCS indicates dietary choline supplementation level in offspring diets (mg/kg). Treatments are expressed as combinations (e.g., E_0_G_0_, E_0.5_G_200_, E_1.5_G_400_). BW = body weight; all other variables are body size traits measured at 28 days of age. SEM = standard error of the mean. For the main-effect means (ECS or GCS), values within the same column with different superscript letters (a-c) differ significantly (*P* < 0.05, FDR-adjusted). *P*-values indicate the effects of ECS, GCS, and their interaction (ECS × GCS) based on FDR-adjusted *P* values.

Table 9 indicates that, at 28 d, heart weight, breast muscle weight, and ovary weight were significantly affected by GCS, with higher values in G_400_ than in G_0_. Liver weight, kidney weight, and bursa of Fabricius weight also differed among GCS treatments; kidney weight was higher in G_400_ than in G_0_, whereas liver weight and bursa of Fabricius weight were higher in G_0_. No significant GCS effects were found for spleen, thymus, or leg muscle weight (*P* > 0.05). ECS only significantly affected bursa of Fabricius weight (*P* = 0.041), with E_0_ being the highest. Significant ECS × GCS interactions were observed for kidney weight, bursa of Fabricius weight, breast muscle weight, and ovary weight (*P* ≤ 0.05).

**Table 9:** Effect of different choline supplementation concentrations in the embryo and its offspring diets on organ indices of goslings at 28 days of age.

Items	ECS (mg/kg)	GCS (mg/kg)	Heart Weight (g)	Liver Weight (g)	Spleen Weight (g)	Kidney Weight (g)	Thymus Weight (g)	Bursa of Fabricius Weight (g)	Pectoralis Muscle Weight (g)	Leg Muscle Weight (g)	Ovary Weight (g)
E_0_G_0_	0	0	9.60	53.83	2.17	14.43	3.440	1.78	18.24	220.72	0.124
E_0_G_200_	0	200	9.18	43.98	1.83	12.66	3.625	1.71	23.88	194.10	0.277
E_0_G_400_	0	400	8.79	43.56	1.95	13.45	3.376	1.05	18.40	163.60	0.297
E_0.5_G_0_	0.5	0	9.20	59.16	2.09	13.60	3.632	1.43	12.66	194.30	0.270
E_0.5_G_200_	0.5	200	8.59	38.53	1.66	11.77	2.393	0.87	21.66	154.54	0.282
E_0.5_G_400_	0.5	400	9.75	47.04	1.74	14.50	4.048	1.22	18.76	182.82	0.270
E_1_G_0_	1.0	0	9.27	52.68	1.89	7.66	3.370	1.44	14.42	160.00	0.150
E_1_G_200_	1.0	200	9.70	42.39	2.07	12.65	3.552	1.31	18.48	200.18	0.165
E_1_G_400_	1.0	400	10.3	46.03	2.69	15.17	3.372	1.36	22.82	218.12	0.515
E_1.5_G_0_	1.5	0	7.85	43.77	1.64	10.51	3.048	1.24	13.26	142.38	0.195
E_1.5_G_200_	1.5	200	9.23	45.38	2.20	11.83	2.257	0.98	16.08	163.68	0.100
E_1.5_G_400_	1.5	400	10.05	45.00	2.69	13.10	2.222	1.36	20.10	230.18	0.460
SEM			0.225	1.000	0.076	0.357	0.146	0.042	0.390	2.791	0.011
	0		9.19	47.03	1.984	13.51	3.480	1.513^a^	20.22	192.74	0.232
	0.5		9.10	48.24	1.831	13.29	3.357	1.172^b^	17.69	177.22	0.274
	1		9.75	47.03	2.214	11.83	3.431	1.371^ab^	18.58	192.76	0.277
	1.5		9.04	44.71	1.972	11.82	2.613	1.192^b^	16.18	178.74	0.252
		0	8.98	52.35^a^	1.950	11.55	3.372	1.473^a^	14.64^b^	179.30	0.185^b^
		200	9.17	42.56^b^	1.938	12.23	3.035	1.217^b^	20.02^a^	178.12	0.206^b^
		400	9.72	45.35^b^	2.114	14.06	3.254	1.246^ab^	20.12^a^	198.86	0.385^a^
*P*-value	ECS		0.332	0.710	0.466	0.305	0.222	0.041	0.143	0.439	0.531
	GCS		0.093	0.003	0.657	0.039	0.703	0.044	0.038	0.121	<0.001
	ECS.GCS interaction		0.064	0.173	0.323	0.050	0.491	0.020	0.003	0.175	<0.001

Note: ECS indicates embryonic choline supplementation level (in ovo injection; mg/mL), and GCS indicates dietary choline supplementation level in offspring diets (mg/kg). Treatments are expressed as combinations (e.g., E_0_G_0_, E_0.5_G_200_, E_1.5_G_400_). Organ indices were calculated as relative organ weight (organ weight/body weight, g/kg). SEM = standard error of the mean. For the main-effect means (ECS or GCS), values within the same column with different superscript letters (a-c) differ significantly (*P* < 0.05, FDR-adjusted). *P*-values indicate the effects of ECS, GCS, and their interaction (ECS × GCS) based on FDR-adjusted *P* values.

### In ovo choline enhances muscle fiber development in goslings

**Fig. 3:**
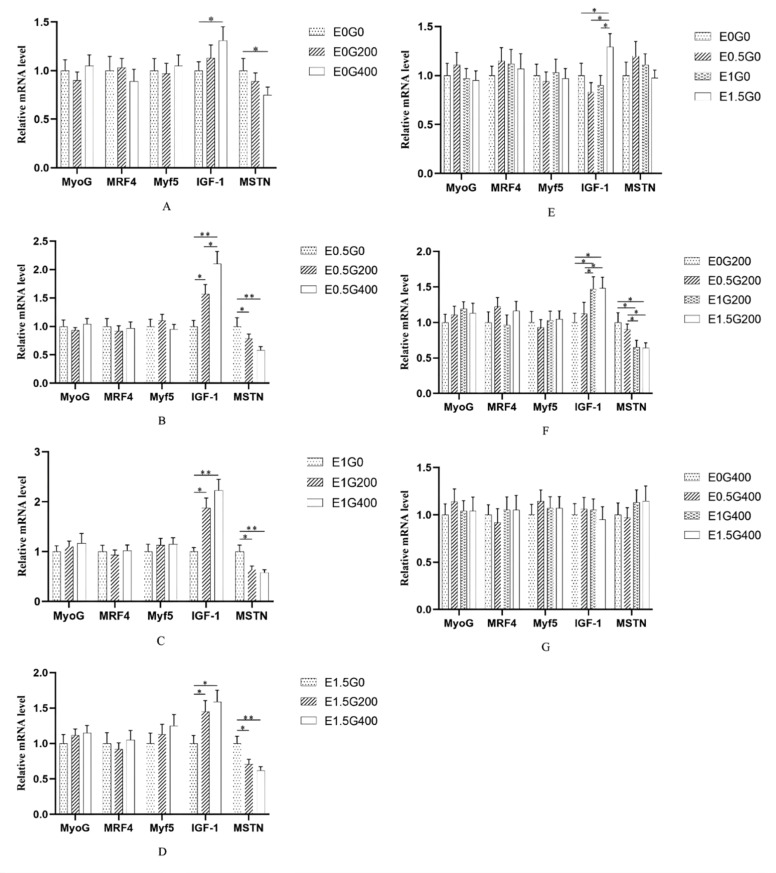
Embryonic and dietary choline modulate pectoralis muscle mRNA expression in goslings at 28 days of age.

Note: Panels A-D present dietary effects within each ECS level (A: E_0_; B: E_0.5_; C: E_1_; D: E_1.5_). Panels E-G present ECS effects within each dietary level (E: G_0_; F: G_200_; G: G_400_). Data are presented as mean ± SEM. Asterisks indicate significant differences between groups (*P < 0.05, **P < 0.01).

**Fig. 4:**
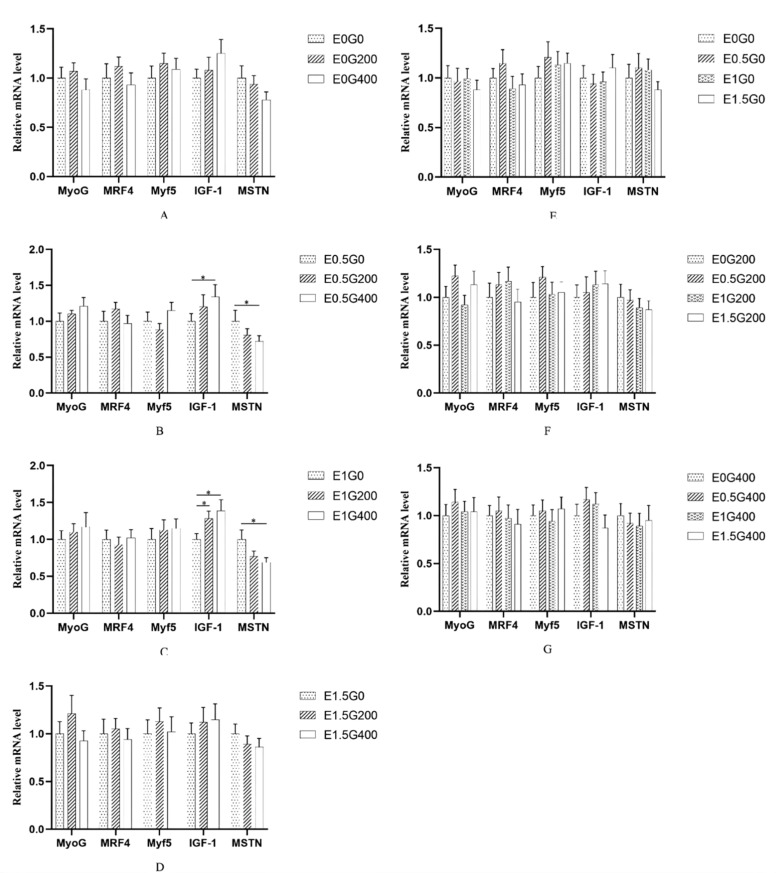
Embryonic and dietary choline modulate leg muscle mRNA expression in goslings at 28 days of age.

Table 10 shows the effect of ECS and GCS on muscle traits at 28 d. For the pectoralis, ECS and GCS significantly affected fiber diameter (*P* ≤ 0.003) and cross-sectional area (*P* ≤ 0.008), with E_1.5_ and G_400_ showing the highest values, while fiber density was lowest (*P* < 0.001). The ECS × GCS interaction was significant for fiber diameter (*P* = 0.048), cross-sectional area (*P* < 0.05), and fiber density (*P* < 0.001). For leg muscle, ECS and GCS significantly affected fiber diameter (*P* ≤ 0.003) and cross-sectional area (*P* ≤ 0.045), with E_1.5_ and G_400_ showing the highest values. The interaction was significant for fiber diameter (*P* = 0.045). *MyoG, MRF4*, and *Myf5* expression did not differ among groups in either muscle (*P* > 0.05). In the pectoralis, *IGF-1* increased and *MSTN* decreased with higher GCS across all ECS backgrounds (*P* < 0.05; Fig. 3A–D). Under the G_0_ background (Fig. 3E), *IGF-1* in E_1.5_G_0_ was higher than in other groups (*P* < 0.05), whereas *MyoG, MRF4, Myf5*, and *MSTN* showed no differences (*P* > 0.05). Under the G_200_ background (Fig. 3F), *IGF-1* in E_0_G_200_ and E_0.5_G_200_ was lower than in E_1_G_200_ and E_1.5_G_200_ (*P* < 0.05), whereas *MSTN* showed the opposite pattern (*P* < 0.05). Under the G_400_ background (Fig. 3G), no significant differences were detected for any gene (*P* > 0.05). In the leg muscle, no significant differences were observed for any gene under most backgrounds (Fig. 4A, 4D, 4E–G; *P* > 0.05), except under the E_0.5_ background (Fig. 4B), where *IGF-1* in E_0.5_G_400_ was higher than in E_0.5_G_200_ (*P* < 0.05), and *MSTN* in E_0.5_G_400_ was lower than in E_0.5_G_0_ (*P* < 0.05), and under the E_1_ background (Fig. 4C), where *IGF-1* in E_1_G_200_ and E_1_G_400_ was higher than in E_1_G_0_ (*P* < 0.05), whereas *MSTN* in E_1_G_0_ was higher than in E_1_G_200_ (*P* < 0.05) and E_1_G_400_ (*P* < 0.01).

**Table 10:** Effect of different choline supplementation concentrations in the embryo and its offspring diets on muscle development of goslings at 28 days of age.

Items	ECS (mg/kg)	GCS (mg/kg)	Pectoralis Muscle			Leg Muscle		
			Fiber diameter (μm)	Fiber cross-sectional area (μm^2^)	Fiber density (fiber/mm^2)^	Fiber diameter (μm)	Fiber cross-sectional area (μm^2^)	Fiber density (fiber/mm^2)^
E_0_G_0_	0	0	2.234	18.24	9578.24	28.44	532.37	1627.06
E_0_G_200_	0	200	2.795	22.95	9553.19	33.59	570.36	1606.98
E_0_G_400_	0	400	4.299	36.53	9610.08	41.50	665.27	1577.73
E_0.5_G_0_	0.5	0	2.012	15.11	9783.43	25.26	501.17	1641.09
E_0.5_G_200_	0.5	200	3.076	25.76	9778.66	35.33	601.50	1588.97
E_0.5_G_400_	0.5	400	4.467	37.07	9821.64	42.28	670.69	1563.87
E_1_G_0_	1.0	0	2.140	16.40	9937.86	27.39	513.97	1615.60
E_1_G_200_	1.0	200	3.865	33.65	9971.04	39.39	646.35	1604.86
E_1_G_400_	1.0	400	4.804	38.72	8443.27	43.96	687.20	1561.11
E_1.5_G_0_	1.5	0	2.850	23.50	9338.80	32.65	584.99	1591.08
E_1.5_G_200_	1.5	200	3.906	34.06	8582.34	39.66	652.02	1571.88
E_1.5_G_400_	1.5	400	4.491	37.37	7011.81	42.44	673.65	1572.77
SEM			0.106	1.615	141.11	2.239	61.55	160.15
	0		3.14^c^	25.91^b^	9580.52^ab^	34.51^b^	589.33^b^	1610.59
	0.5		3.18^bc^	25.98^b^	9794.58^a^	34.29^b^	591.12^ab^	1607.64
	1		3.60^ab^	29.59^ab^	9450.73^b^	36.91^ab^	615.84^ab^	1593.86
	1.5		3.75^a^	31.64^a^	8310.98^c^	38.25^a^	636.88^a^	1580.58
		0	2.33^c^	18.31^c^	9659.60^a^	28.44^c^	533.13^c^	1613.71
		200	3.41^b^	29.10^b^	9471.31^a^	36.99^b^	617.56^b^	1593.17
		400	4.51^a^	37.42^a^	8721.70^b^	42.54^a^	674.20^a^	1571.45
*P*-value	ECS		0.003	0.008	<0.001	0.003	0.041	0.334
	GCS		<0.001	<0.001	<0.001	<0.001	<0.001	0.089
	ECS.GCS interaction		0.048	0.047	<0.001	0.045	0.286	0.565

Note: ECS indicates embryonic choline supplementation level (in ovo injection; mg/mL), and GCS indicates dietary choline supplementation level in offspring diets (mg/kg). Treatments are expressed as combinations (e.g., E_0_G_0_, E_0.5_G_200_, E_1.5_G_400_). SEM = standard error of the mean. For the main-effect means (ECS or GCS), values within the same column with different superscript letters (a-c) differ significantly (*P* < 0.05, FDR-adjusted). *P*-values indicate the effects of ECS, GCS, and their interaction (ECS × GCS) based on FDR-adjusted *P* values.

Panels A-D present dietary effects within each ECS level (A: E_0_; B: E_0.5_; C: E_1_; D: E_1.5_). Panels E-G present ECS effects within each dietary level (E: G_0_; F: G_200_; G: G_400_). Data are presented as mean ± SEM. Asterisks indicate significant differences between groups (*P < 0.05, **P < 0.01).

### In ovo choline modulates hormonal profiles in goslings

Table 11 shows that, at 7 d, ECS significantly affected IGF-1, GH, CORT, and ACT (*P* < 0.001). GCS significantly affected IGF-1, T3, T4, GH, CORT, and ACT (*P* < 0.001). The ECS × GCS interaction was significant for IGF-1 and CORT (*P* ≤ 0.041). At 28 d, ECS did not significantly influence any hormone (*P* > 0.05), whereas GCS significantly affected IGF-1, T3, T4, GH, CORT, and ACT (*P* ≤ 0.047). The ECS × GCS interaction was not significant (*P* > 0.05).

**Table 11:** Effect of different choline supplementation concentrations in the embryo and its offspring diets on blood hormone and biochemical indices were measured at 7 and 28 days of age.

Items	ECS (mg/kg)	GCS (mg/kg)	Age(d)	IGF-1 (ng/ml)	T_3_ (nmol/L)	T_4_ (nmol/L)	GH (ng/ml)	CORT (pg/ml)	ACT (ng/ml)
E_0_G_0_	0	0	7	86.5	3.18	116.8	9.82	15.82	3.58
E_0_G_200_	0	200		89.2	4.93	141.6	10.41	15.24	3.42
E_0_G_400_	0	400		88.6	4.05	123.9	11.75	14.65	3.26
E_0.5_G_0_	0.5	0		86.4	3.27	118.1	12.76	14.53	3.31
E_0.5_G_200_	0.5	200		92.4	4.87	142.7	13.98	13.42	3.15
E_0.5_G_400_	0.5	400		91.6	4.11	125.1	15.20	12.87	2.99
E_1_G_0_	1.0	0		80.8	3.22	117.0	15.42	13.21	3.05
E_1_G_200_	1.0	200		83.7	4.88	140.9	16.60	12.03	2.89
E_1_G_400_	1.0	400		94.0	3.97	124.3	17.90	10.82	2.73
E_1.5_G_0_	1.5	0		80.7	3.32	118.7	15.35	13.12	3.04
E_1.5_G_200_	1.5	200		82.6	4.96	142.4	16.58	12.14	2.88
E_1.5_G_400_	1.5	400		96.9	4.09	125.6	17.85	10.73	2.72
E_0_G_0_	0	0	28	109.7	4.69	157.0	17.20	24.23	3.92
E_0_G_200_	0	200		110.5	7.05	200.4	19.22	22.61	3.65
E_0_G_400_	0	400		112.5	5.68	171.8	21.65	20.92	3.38
E_0.5_G_0_	0.5	0		107.5	4.81	158.1	17.84	23.41	3.81
E_0.5_G_200_	0.5	200		113.1	7.12	201.2	20.11	21.79	3.54
E_0.5_G_400_	0.5	400		114.7	5.77	172.9	22.58	20.11	3.27
E_1_G_0_	1.0	0		112.5	4.73	157.1	18.20	22.52	3.70
E_1_G_200_	1.0	200		113.4	6.98	199.9	20.75	20.91	3.43
E_1_G_400_	1.0	400		114.4	5.71	172.0	23.30	19.23	3.16
E_1.5_G_0_	1.5	0		108.3	3.32	158.4	18.25	22.43	3.69
E_1.5_G_200_	1.5	200		111.4	4.96	201.6	20.80	20.83	3.42
E_1.5_G_400_	1.5	400		117.4	4.09	173.6	23.35	19.15	3.15
SEM			7	7.40	0.46	10.40	2.40	1.52	0.30
			28	10.61	0.63	19.10	3.44	2.53	0.35
	0		7	88.1^b^	4.05	127.4	10.49^c^	14.92^a^	3.42^a^
	0.5			90.1^a^	4.10	128.6	13.98^b^	13.61^b^	3.15^b^
	1			86.2^c^	4.02	127.4	16.64^a^	12.09^c^	2.89^c^
	1.5			86.7^c^	4.12	128.9	16.59^a^	12.01^c^	2.88^c^
	0		28	110.9	5.81	176.4	19.36	22.34	3.65
	0.5			111.8	5.90	177.4	20.18	21.83	3.54
	1			113.4	5.81	176.3	20.75	21.02	3.43
	1.5			112.4	5.94	177.7	20.80	20.91	3.42
		0	7	83.6^c^	3.20^c^	117.4^c^	14.08^c^	13.71^a^	3.25^a^
		200		87.0^b^	4.91^a^	141.7^b^	15.32^b^	12.89^b^	3.09^b^
		400		92.8^a^	4.06^b^	124.6^a^	16.63^a^	11.97^c^	2.91^c^
		0	28	109.3^b^	4.75^c^	157.4^c^	17.90^c^	22.47^a^	3.73^a^
		200		111.9^b^	7.05^a^	200.5^b^	20.27^b^	21.03^b^	3.46^b^
		400		114.5^a^	5.76^b^	173.2^a^	22.65^a^	19.52^c^	3.19^c^
*P*-value	ECS		7	<0.001	0.521	0.511	<0.001	<0.001	<0.001
	GCS			<0.001	<0.001	<0.001	<0.001	<0.001	<0.001
	ECS.GCS interaction			<0.001	0.884	0.840	0.282	0.041	0.335
			28	0.455	0.487	0.468	0.191	0.334	0.463
				0.047	<0.001	<0.001	<0.001	<0.001	<0.001
				0.641	0.830	0.807	0.318	0.426	0.335

Note: ECS indicates embryonic choline supplementation level (in ovo injection; mg/mL), and GCS indicates dietary choline supplementation level in offspring diets (mg/kg). Treatments are expressed as combinations (e.g., E_0_G_0_, E_0.5_G_200_, E_1.5_G_400_). SEM = standard error of the mean. For the main-effect means (ECS or GCS), values within the same column with different superscript letters (a-c) differ significantly (*P* < 0.05, FDR-adjusted). *P*-values indicate the effects of ECS, GCS, and their interaction (ECS × GCS) based on FDR-adjusted *P* values.

## Discussion

### Stage-dependent effects of in ovo choline on embryo growth at DE18 and DE27

Sham-injected embryos (E_PC_; 14.61 g) weighed less than non-injected controls (E_NC_; 18.53 g) at DE18, yet this difference disappeared by DE27 (70.03 g vs. 70.65 g), indicating that the injection procedure induced a transient stress response rather than sustained damage. Because all choline-supplemented groups received the identical injection protocol, comparisons against the E_PC_ baseline remain internally valid. Nevertheless, the acute stress associated with in ovo injection represents a potential confounding factor for early endpoints, and future studies should consider non-invasive maternal dietary strategies to eliminate this bias. At DE18, choline supplementation significantly increased embryo weight and relative embryo weight. Because DE18 represents a period of rapid growth and intensive cell proliferation, choline is essential for supporting these processes through its roles in membrane biosynthesis, methyl-group metabolism, and energy-related pathways (Huang et al., 2023). However, no significant effects were observed at DE20 or DE23, possibly because embryonic growth becomes relatively stable during these stages, major developmental programs are already established, and nutritional demands change less markedly. By DE27, metabolic requirements increase and buffering capacity declines, which may amplify phenotypic differences and lead to the re-emergence of significant effects. In a mouse model of placental insufficiency, higher choline increased embryo weight at embryonic day 10.5, whereas differences were not significant at embryonic days 12.5-15.5; moreover, at embryonic day 18.5, supplementation with 5.6 g choline chloride/kg diet resulted in a significant decrease in embryo weight compared with 1.4 g choline chloride/kg diet (King et al., 2017). These patterns indicate that choline effects may increase linearly within a certain dosage range, while for some hormones, increasing choline may trigger negative-feedback regulation, resulting in saturation or attenuation of responses (Ritchie, 2014; Liu et al., 2025). Overall, the effect of choline supplementation on embryo weight appeared to be window-specific, being more evident at DE18 (high proliferative demand) and DE27 (high metabolic load close to hatch), but less apparent at DE20–DE23, when developmental programs are relatively stable.

### Interactive effects of in ovo and dietary choline on gosling growth and development

#### Growth performance and feed conversion efficiency

A significant main effect of ECS was already observed at 1 day of age (with the E_1_ group showing a higher BW), suggesting that in ovo choline injection may be reflected in hatch weight through differences in body nutrient reserves and/or tissue maturity during late incubation (Gouda et al., 2022). Previous studies have reported that interactions between embryonic nutritional interventions and post-hatch nutrient supply are a typical feature of early-life nutritional programming (Jha et al., 2019; Das et al., 2021). By 7 days of age, the ECS main effect remained significant and a significant ECS × GCS interaction had emerged, indicating that the embryonic choline background can modify the magnitude of goslings’ responses to subsequent dietary choline levels. Because in-egg nutrient reserves may become relatively limited during late embryogenesis, supplying key nutrients at this stage can influence embryonic growth as well as post-hatch intestinal development, immunity, and productive performance (Jha et al., 2019). In broilers, an in ovo nutrient supplementation × early/late access-to-feed design showed that the promotive effects of in ovo feeding on post-hatch BW and muscle development were more pronounced under early feeding, further supporting that embryonic interventions can alter growth rates under different post-hatch nutritional conditions (Kornasio et al., 2011). From 14 to 28 days of age, the main effect of GCS became evident and, together with ECS, influenced BW; moreover, the interaction effect remained significant throughout the experimental period. These findings indicate that the embryonic choline background not only affects the baseline BW but also modulates the efficacy of later dietary choline supplementation. Similar in ovo nutrition × post-hatch dietary supplementation interactions have been reported in two-stage nutritional studies; for example, an in ovo × dietary betaine combination produced significant interactive effects on growth performance during 7-21 days of age (Dadvar et al., 2022). Overall, the persistent ECS–GCS interaction observed here is consistent with the characteristic “early-late” nutritional interplay described in nutritional programming and two-stage nutrition research, highlighting that choline supplementation strategies should consider coordination between the embryonic and brooding phases to achieve optimal growth outcomes.

During 1–14 days of age, ADFI was not affected by ECS or GCS. ADG and F/G showed a significant main effect of GCS, with no significant ECS × GCS interaction. This pattern suggests that the growth-promoting influence of choline was primarily manifested as an increased growth rate rather than altered feed intake. Consistent with previous poultry studies, nutritional interventions involving choline or other methyl donors tend to exert more sensitive effects on growth performance and feed efficiency than on feed intake, and responses may not be strictly linear across doses or delivery forms (Gholami et al., 2015; Ramalho De Lima et al., 2024). During 15–28 days of age, ECS significantly affected ADFI and F/G, and GCS also improved ADG and F/G; moreover, the ECS × GCS interaction was significant for ADFI and F/G. The perihatch and early post-hatch period represents a critical window for rapid development of the digestive tract and nutrient absorption capacity, and in ovo nutritional interventions may shift subsequent trajectories of nutrient utilization and productive performance, thereby leading to differential responses under different later dietary nutrient levels (Jha et al., 2019; Das et al., 2021). This interpretation is also consistent with the sustained BW interaction observed in Table 6. In addition, previous work using combined methyl-donor strategies across in ovo and dietary phases reported significant interactions in growth traits, providing further support for the persistent ECS × GCS interactions on ADFI and F/G observed in the present study (Dadvar et al., 2022). Collectively, the effects of choline on gosling growth were phase-dependent: during 1-14 days, choline mainly increased ADG and improved F/G without altering ADFI; during 15-28 days, the main effects and interactions of ECS and GCS became more apparent, suggesting that embryonic choline background may, via early nutritional programming, regulate subsequent utilization of dietary choline and growth efficiency, in agreement with the sustained BW interaction across the entire period.

#### Body size traits and organ development

Based on the results, GCS significantly affected body weight, half-submerged length, and keel length, which may be attributable to higher choline availability supporting energy metabolism, protein synthesis, and muscle development (Arif et al., 2022). In contrast, GCS had no significant effects on other body size traits such as neck length, body length, and shank circumference, suggesting that these skeletal characteristics are less sensitive to choline supply or are more strongly constrained by genetic factors and the overall balance of other dietary nutrients. No significant differences were detected among ECS groups for these morphometric indices, indicating that embryonic nutritional intervention may primarily influence early organogenesis and that its effects at 28 days of age could be masked by later nutrition and growth or compensated during post-hatch development. The ECS × GCS interaction may contribute to BW gain by coordinating fatty acid synthesis and storage as well as muscle and skeletal growth (Moretti et al., 2020). The interactive effects observed for traits such as shank circumference and hip width may reflect the complementary roles of embryonic and dietary choline during skeletal development, potentially through improved nutrient utilization efficiency and enhanced mineral deposition in bone (Øyen et al., 2017).

At the organ level, the G_400_ group exhibited significantly greater heart, pectoralis muscle, and ovary weights than the G_0_ group, and significant interaction effects were detected for the weights of the heart, kidney, bursa of Fabricius, pectoralis muscle, and ovary. These findings are consistent with the increased BW observed in Table 8, indicating that choline supplementation was accompanied by changes in selected tissue and organ traits. The increased pectoralis muscle weight may be related to the role of choline in promoting muscle protein synthesis and myofiber development (Gao et al., 2026). Under GCS treatments, kidney weight increased with increasing choline level, whereas liver weight and bursa of Fabricius weight were higher in the absence of dietary choline; under ECS treatments, only the bursa of Fabricius showed a significant main effect, with the highest value in E_0_. As choline participates in one-carbon metabolism and phospholipid synthesis, thereby influencing lipid transport, methylation reactions, and antioxidant capacity, the liver and kidney, key metabolic and excretory organs, may differ in their sensitivity to choline supply (May et al., 2018; Gong et al., 2023; Kenny et al., 2025). While reduced liver weight may reflect improved metabolic efficiency or decreased hepatic lipid accumulation under higher choline supply. Moreover, the bursa of Fabricius is a critical immune organ whose development during embryogenesis may be regulated by choline availability, thereby influencing the establishment of immune function (Maggini et al., 2018; Munteanu and Schwartz, 2022). The lower bursa of Fabricius weight at G400 raises a cautionary note. The bursa is the primary site of B-cell development in poultry, and its reduced mass at 28 days may indicate altered immune organ development rather than a purely beneficial outcome. We acknowledge that this could impair long-term humoral immunity, though functional immune endpoints were not assessed here. Future studies should examine antibody responses to clarify whether high-dose choline entails an immune trade-off.

Overall, GCS exerted significant effects on gosling growth and visceral organ development, with higher choline level (G_400_) being associated with improved BW and selected skeletal development indices. The effects of GCS were mainly manifested in BW, skeletal traits, visceral organ weights, and muscle growth, whereas ECS had comparatively limited influence on these endpoints, suggesting that embryonic choline may play a more prominent role in other developmental processes (Norris et al., 2022).

### Synergistic effects of in ovo and dietary choline on muscle fiber development and IGF-1/MSTN mRNA expression

Choline is an essential nutrient for protein metabolism. As a methyl donor, this micronutrient influences protein homeostasis by promoting protein synthesis and reducing protein degradation (Zhang et al., 2024). In poultry, the number of skeletal muscle fibers is largely determined before hatch; therefore, nutrient supply from the yolk is critical for myogenesis (Muyyarikkandy et al., 2023). In the present study, both ECS and GCS significantly affected myofiber diameter and cross-sectional area in the pectoral and leg muscles of 28-day-old goslings, with the E_1.5_ and G_400_ groups showing the most favorable muscle traits. Consistently, supplementing breeder hen diets with 900 mg/kg choline markedly increased the number of pectoralis muscle fibers in embryos, enhanced myoblast proliferation, and upregulated the expression of myogenic genes such as *MyoD* (Gao et al., 2026). Notably, choline enhanced myofiber hypertrophy without altering the classical myogenic factors *MyoG, MRF4*, or *Myf5* in either muscle type, indicating that the effect is not mediated through the MyoD-family pathway. Instead, the coordinated upregulation of *IGF-1* and downregulation of *MSTN* in the pectoralis muscle suggest that choline promotes hypertrophy primarily through the IGF-1/MSTN axis. The weaker transcriptional response in the leg muscle, despite comparable morphological gains, indicates muscle-type-specific differences in molecular sensitivity; therefore, the pectoralis molecular profile should not be generalized to all skeletal muscles. Dietary choline supplementation has also been reported to increase serum IGF2 levels; IGF2 regulates fetal growth, promotes amino acid and glucose uptake, and reduces *IGFBP-2* levels in skeletal muscle fibers (Oster et al., 2016; Tysoe, 2024). In addition, choline is involved in regulating membrane biosynthesis, lipid metabolism, and methylation reactions during embryogenesis, processesing that are crucial for myoblast proliferation and differentiation (Mikołajczyk-Stecyna et al., 2024). Moreover, by altering DNA methylation, predominantly toward hypomethylation), choline supplementation can exert long-term effects on postnatal body weight, weight gain, and muscle development (Estrada-Cortés et al., 2021).

### Regulation of the GH/IGF-1 axis, thyroid hormones, and corticosterone by in ovo and dietary choline

ECS, particularly the E_1_ treatment, significantly affected circulating concentrations of IGF-1, T_3_, T_4_, GH, and CORT. Previous evidence indicates that embryonic choline availability can influence developmental processes and exert persistent effects on later physiological functions. Choline is a precursor for the synthesis of the neurotransmitter acetylcholine (ACh) (Jahanian and Ashnagar, 2018; Gao et al., 2026), and ACh deficiency has been shown to markedly reduce serum GH, IGF-1, and GnRH levels in mouse embryos (Lecomte et al., 2018). In addition, embryonic betaine injection significantly increased IGF-1 concentration and *IGF-1* mRNA expression in newly hatched goslings (Ma et al., 2024). In the present study, the E_1_ group exhibited the highest hormonal levels, which may be attributable to enhanced IGF-1 synthesis at an appropriate choline dose; IGF-1 plays a pivotal role in cell proliferation, muscle growth, and skeletal development (Estrada-Cortés et al., 2021). Further studies have suggested that choline, by modulating embryonic metabolism, may optimize hormonal regulation during subsequent growth. Excessive CORT is known to impair muscle growth by altering protein synthesis and lipid metabolism, thereby inhibiting key processes required for muscle hypertrophy (Abobaker et al., 2017; Sato et al., 2020). Notably, at 7 days of age, the ECS × GCS interaction was significant for both IGF-1 and CORT, indicating that an appropriate choline supply may enhance stress tolerance and support maturation of the GH/IGF-1 axis, underscoring the importance of choline across both embryonic and early post-hatch stages (Yu et al., 2018). Existing studies further suggest that combined choline supplementation during embryogenesis and the gosling period may act synergistically to optimize endocrine regulation and thereby improve early growth and development. Higher GCS may increase growth potential of the skeleton and musculature, possibly through elevating GH levels. Consistently, maternal choline supplementation during gestation in sows significantly increased GH, T_3_, and T_4_ concentrations in offspring piglets and promoted muscle protein synthesis and myocyte proliferation (Jin et al., 2018; He et al., 2020). Moreover, during late embryogenesis, ACT driven corticosterone elevation stimulates growth hormone secretion, which is critical for pre-hatch growth acceleration and metabolic preparation (Tong et al., 2013). Collectively, the combined effects of ECS and GCS may enhance growth potential by regulating IGF-1 and GH, while modulating T_3_, T_4_, and CORT to improve metabolic rate and stress adaptability. These findings suggest that choline acts during both the embryonic and post-hatch periods, and that their interaction may generate amplified endocrine benefits.

## Conclusion

0.5 mL of a 1.0 mg/mL in-ovo choline solution increased embryo weight at embryonic day 18 and 27. Adding 400 mg/kg dietary choline significantly increased body weight at 28 days, improved FCR, and enhanced muscle fiber diameter and cross-sectional area in pectoralis muscles. These improvements were associated with elevated embryonic GH, ACT, increased serum IGF-1, and pectoral IGF-1/MSTN axis modulation, indicating that choline promotes muscle growth via the IGF-1/MSTN axis. Optimal choline levels depend on the production target. For growth rate and feed efficiency, E_1_G_400_ is preferable; for muscle yield, E_1.5_G_400_ offers additional fiber gains. For immune organ development, E_0_G_0_ is preferable given the increased bursa weights at lower supplementation.

## Funding

This work was financially supported by the Project of the China Agriculture Research System (CARS 42-11).

## Institutional review board statement

Not applicable.

## Informed consent statement

Informed consent was obtained from all subjects involved in the study.

## Data availability statement

The data presented in this study are available on request from the corresponding author.

## CRediT authorship contribution statement

**Wenfeng Liu:** Conceptualization, Methodology, Investigation, Data curation, Formal analysis, Writing – original draft, Writing – review & editing. **Xucheng Zheng:** Validation, Writing – review & editing. **Xuan Li:** Software, Visualization, Writing – review & editing. **Haiming Yang:** Supervision, Writing – review & editing. **Zhiyue Wang:** Supervision, Project administration, Funding acquisition, Conceptualization, Writing – review & editing.

## Disclosures

The authors declare no conflict of interest.

## References

[bib0001] Abobaker H., Hu Y., Hou Z., Sun Q., Idriss A.A., Omer N.A., Zong Y., Zhao R. (2017). Dietary betaine supplementation increases adrenal expression of steroidogenic acute regulatory protein and yolk deposition of corticosterone in laying hens. Poult. Sci..

[bib0002] Abobaker H., Omer N.A., Hu Y., Idriss A.A., Zhao R. (2022). In ovo injection of betaine promotes adrenal steroidogenesis in pre-hatched chicken fetuses. Poult. Sci..

[bib0003] Arif M., Baty R.S., Althubaiti E.H., Ijaz M.T., Fayyaz M., Shafi M.E., Albaqami N.M., Alagawany M., Abd El-Hack M.E., Taha A.E., Salem H.M., El-Tahan A.M., Elnesr S.S. (2022). The impact of betaine supplementation in quail diet on growth performance, blood chemistry, and carcass traits. Saudi J. Biol. Sci..

[bib0004] Beheshti Moghadam M.H., Aziza A.E., Cherian G. (2021). Choline and methionine supplementation in layer hens fed flaxseed: effects on hen production performance, egg fatty acid composition, tocopherol content, and oxidative stability. Poult. Sci..

[bib0005] Bhattacharyya A., Majumdar S., Bhanja S.K., Mandal A.B., Kadam M.M. (2017). Effect of maternal dietary manipulation and in ovo injection of nutrients on the body weight gain, feed conversion ratio, development of lymphoid and digestive organs of turkey poults. Indian J. Anim. Sci..

[bib0006] Burns B.C., Belani J.D., Wittorf H.N., Brailoiu E., Brailoiu G.C. (2025). Choline–An essential nutrient with health benefits and a signaling molecule. Int. J. Mol. Sci..

[bib0007] Conerly M.L., Yao Z., Zhong J.W., Groudine M., Tapscott S.J. (2016). Distinct activities of Myf5 and MyoD indicate separate roles in skeletal muscle lineage specification and differentiation. Dev. Cell.

[bib0008] Dadvar P., Maddahian A., Dayani O. (2022). In ovo and dietary feeding of betaine to broiler chickens under heat stress conditions: effects on hatchability, performance, body temperature and blood parameters. J. Livest. Sci. Technol. (JLST).

[bib0009] Das R., Mishra P., Jha R. (2021). In ovo feeding as a tool for improving performance and gut health of poultry: a review. Front. Vet. Sci..

[bib0010] Estrada-Cortés E., Hansen P.J. (2020). 54 choline alters the pattern of DNA methylation and lipid content of pre-implantation bovine embryos. Reprod. Fertil. Dev..

[bib0011] Estrada-Cortés E., Ortiz W., Rabaglino M.B., Block J., Rae O., Jannaman E.A., Xiao Y., Hansen P.J. (2021). Choline acts during preimplantation development of the bovine embryo to program postnatal growth and alter muscle DNA methylation. FASEB J..

[bib0012] Gao M., Han Q., Zhu Q., Li D., Li X., Lv Z., Guo Y. (2026). Maternal effects of choline on skeletal muscle development and intramuscular fat deposition in broiler offspring. Poult. Sci..

[bib0013] Gholami J., Qotbi A.A.A., Seidavi A., Meluzzi A., Tavaniello S., Maiorano G. (2015). Effects of in *ovo* administration of betaine and choline on hatchability results, growth and carcass characteristics and immune response of broiler chickens. Ital. J. Anim. Sci..

[bib0014] Gong M., Lu H., Li L., Feng M., Zou Z. (2023). Integration of transcriptomics and metabonomics revealed the protective effects of hemp seed oil against methionine–choline-deficient diet-induced non-alcoholic steatohepatitis in mice. Food Funct..

[bib0015] Gouda A., Tolba S.A., Mahrose K.M. (2022). Influences of vitamin a, L-carnitine, and folic acid *in ovo* feeding on embryo and hatchling characteristics and general health status in ducks. Anim. Biotechnol..

[bib0016] He Q., Zou T., Chen J., Jian L., He J., Xia Y., Xie F., Wang Z., You J. (2020). Maternal methyl-donor micronutrient supplementation during pregnancy promotes skeletal muscle differentiation and maturity in newborn and weaning pigs. Front. Nutr..

[bib0017] Huang B., Khan M.Z., Kou X., Chen Y., Liang H., Ullah Q., Khan N., Khan A., Chai W., Wang C. (2023). Enhancing metabolism and milk production performance in periparturient dairy cattle through rumen-protected methionine and choline supplementation. Metabolites.

[bib0018] Jahanian R., Ashnagar M. (2018). Effects of dietary supplementation of choline and carnitine on growth performance, meat oxidative stability and carcass composition of broiler chickens fed diets with different metabolisable energy levels. Br. Poult. Sci..

[bib0019] Jha R., Singh A.K., Yadav S., Berrocoso J.F.D., Mishra B. (2019). Early nutrition programming (in ovo and post-hatch feeding) as a strategy to modulate gut health of poultry. Front. Vet. Sci..

[bib0020] Jin C., Zhuo Y., Wang J., Zhao Y., Xuan Y., Mou D., Liu H., Zhou P., Fang Z., Che L., Xu S., Feng B., Li J., Jiang X., Lin Y., Wu D. (2018). Methyl donors dietary supplementation to gestating sows diet improves the growth rate of offspring and is associating with changes in expression and DNA methylation of insulin-like growth factor-1 gene. J. Anim. Physiol. Anim. Nutr..

[bib0021] Kenny T.C., Scharenberg S., Abu-Remaileh M., Birsoy K. (2025). Cellular and organismal function of choline metabolism. Nat. Metab..

[bib0022] King J., Kwan S., Yan J., Klatt K., Jiang X., Roberson M., Caudill M. (2017). Maternal choline supplementation alters fetal growth patterns in a mouse model of placental insufficiency. Nutrients.

[bib0023] Kornasio R., Halevy O., Kedar O., Uni Z. (2011). Effect of in ovo feeding and its interaction with timing of first feed on glycogen reserves, muscle growth, and body weight. Poult. Sci..

[bib0024] Lecomte M.J., Bertolus C., Ramanantsoa N., Saurini F., Callebert J., Sénamaud-Beaufort C., Ringot M., Bourgeois T., Matrot B., Collet C., Nardelli J., Mallet J., Vodjdani G., Gallego J., Launay J.M., Berrard S. (2018). Acetylcholine modulates the hormones of the growth hormone/insulinlike growth factor-1 axis during development in mice. Endocrinology.

[bib0025] Lee J., Kim D.H., Lee K. (2024). Myostatin gene role in regulating traits of poultry species for potential industrial applications. J. Anim. Sci. Biotechnol..

[bib0026] Li K., Jiang L., Wang J., Xia L., Zhao R., Cai C., Wang P., Zhan X., Wang Y. (2020). Maternal dietary supplementation with different sources of selenium on antioxidant status and mortality of chicken embryo in a model of diquat-induced acute oxidative stress. Anim. Feed Sci. Technol..

[bib0027] Liu W., Wang J., Wang J., Ji R., Wang Z., Yang H. (2025). Effects of choline on reproductive performance, egg quality, and ovarian morphological development in laying geese. Anim. Feed Sci. Technol..

[bib0028] Ma S., Liu J., Zhao Y., Wang Y., Zhao R. (2024). ovo betaine injection improves breast muscle growth in newly hatched goslings through FXR/IGF-2 pathway. Poult. Sci..

[bib0029] Maggini S., Pierre A., Calder P.C., Maggini S., Pierre A., Calder P.C. (2018). Immune function and micronutrient requirements change over the life course. Nutrients.

[bib0030] May T., Klatt K.C., Smith J., Castro E., Manary M., Caudill M.A., Jahoor F., Fiorotto M.L., May T., Klatt K.C., Smith J., Castro E., Manary M., Caudill M.A., Jahoor F., Fiorotto M.L. (2018). Choline supplementation prevents a hallmark disturbance of kwashiorkor in weanling mice fed a maize vegetable diet: hepatic steatosis of undernutrition. Nutrients.

[bib0031] Mikołajczyk-Stecyna J., Zuk E., Chmurzynska A., Blatkiewicz M., Jopek K., Rucinski M. (2024). The effects of exposure to and timing of a choline-deficient diet during pregnancy and early postnatal life on the skeletal muscle transcriptome of the offspring. Clin. Nutr..

[bib0032] Moretti A., Paoletta M., Liguori S., Bertone M., Toro G., Iolascon G. (2020). Choline: an essential nutrient for skeletal muscle. Nutrients.

[bib0033] Munteanu C., Schwartz B. (2022). The relationship between nutrition and the immune system. Front. Nutr..

[bib0034] Muyyarikkandy M.S., Schlesinger M., Ren Y., Gao M., Liefeld A., Reed S., Amalaradjou M.A. (2023). In ovo probiotic supplementation promotes muscle growth and development in broiler embryos. Poult. Sci..

[bib0035] Norris S.A., Frongillo E.A., Black M.M., Dong Y., Fall C., Lampl M., Liese A.D., Naguib M., Prentice A., Rochat T. (2022). Nutrition in adolescent growth and development. Lancet.

[bib49] NRC (National Research Council) (1994). Nutrient Requirements of Poultry: Ninth Revised.

[bib0036] Osman R.H., Liu L., Xia L., Zhao X., Wang Q., Sun X., Zhang Y., Yang B., Zheng Y., Gong D. (2016). Fads1 and 2 are promoted to meet instant need for long-chain polyunsaturated fatty acids in goose fatty liver. Mol. Cell. Biochem..

[bib0037] Oster M., Nuchchanart W., Trakooljul N., Murani E., Zeyner A., Wirthgen E., Hoeflich A., Ponsuksili S., Wimmers K. (2016). Methylating micronutrient supplementation during pregnancy influences foetal hepatic gene expression and IGF signalling and increases foetal weight. Eur. J. Nutr..

[bib0038] Øyen J., Gjesdal C.G., Karlsson T., Svingen G.F., Tell G.S., Strand E., Drevon C.A., Vinknes K.J., Meyer K., Ueland P.M., Nygård O. (2017). Dietary choline intake is directly associated with bone mineral density in the Hordaland health study. J. Nutr..

[bib0039] Ramalho De Lima M., Kaneko I.N., De Lima A.V., De Melo L.N., De Lima M.C., De Brito A.N.E.F., Costa F.G.P., Boas A.D.C.V., Toledo A.L., Ferrer S.L., Marimuthu S., Tomaszewska E. (2024). Choline supplementation: impact on broiler chicken performance, steatosis, and economic viability from from 1 to 42 days. PLOS ONE.

[bib0040] Ritchie M. (2014). Neuroanatomy and physiology of the avian hypothalamic/pituitary axis. Vet. Clin. N. Am. Exot. Anim. Pract..

[bib0041] Sato M., Sugiyama K., Maeda N., Fujiki J., Ieko T., Kawamura Y., Iwano H., Mukai K., Yokota H. (2020). Local biosynthesis of corticosterone in rat skeletal muscle. J. Steroid Biochem. Mol. Biol..

[bib0042] Tako E., Ferket P.R., Uni Z. (2004). Effects of in ovo feeding of carbohydrates and beta-hydroxy-beta-methylbutyrate on the development of chicken intestine. Poult. Sci..

[bib0043] Tong Q., Romanini C.E., Exadaktylos V., Bahr C., Berckmans D., Bergoug H., Eterradossi N., Roulston N., Verhelst R., McGonnell I.M. (2013). Embryonic development and the physiological factors that coordinate hatching in domestic chickens. Poult. Sci..

[bib0044] Tysoe O. (2024). IGF2-produced microRNA restricts growth via suppression of IGF1. Nat. Rev. Endocrinol..

[bib0045] Xun Y., Jiang Y., Khalid A., Tian Z., Rios J., Zhang Z. (2025). KBTBD2 controls bone development by regulating IGF-1 signaling during osteoblast differentiation. Cell Death Differ..

[bib0046] Yu J., Yan L., Chen Z., Li H., Zhu H., Chen R., Shi Z. (2018). Corticosterone induces growth hormone expression in pituitary somatotrophs during goose embryonic development. J. Reprod. Dev..

[bib0047] Zhang J., Geng S., Zhu Y., Li L., Zhao L., Ma Q., Huang S. (2024). Effects of dietary methionine supplementation on the growth performance, immune responses, antioxidant capacity, and subsequent development of layer chicks. Poult. Sci..

[bib0048] Zielińska-Dawidziak M., Klimowicz P., Tomczak A. (2025). Super eggs production – the influence of feed modification on designer egg composition–a review. Ann. Anim. Sci..

